# Thickness-Dependent Photoelectrochemical Water Splitting Properties of Self-Assembled Nanostructured LaFeO_3_ Perovskite Thin Films

**DOI:** 10.3390/nano11061371

**Published:** 2021-05-21

**Authors:** Florin Andrei, Valentin Ion, Ruxandra Bîrjega, Maria Dinescu, Nicoleta Enea, Dan Pantelica, Maria Diana Mihai, Valentin-Adrian Maraloiu, Valentin Serban Teodorescu, Ioan-Cezar Marcu, Nicu Doinel Scarisoreanu

**Affiliations:** 1 National Institute for Laser, Plasma and Radiation Physics, 077125 Magurele, Romania; florin.andrei@inflpr.ro (F.A.); valentin.ion@inflpr.ro (V.I.); ruxandra.birjega@inflpr.ro (R.B.); maria.dinescu@inflpr.ro (M.D.); nicoleta.enea@inflpr.ro (N.E.); 2 Laboratory of Chemical Technology & Catalysis, Department of Organic Chemistry, Biochemistry & Catalysis, Faculty of Chemistry, University of Bucharest, Blv. Regina Elisabeta, No. 4-12, 030018 Bucharest, Romania; 3 Faculty of Physics, University of Bucharest, 077125 Magurele, Romania; 4 Horia Hulubei National Institute for R&D in Physics and Nuclear Engineering, 077125 Magurele, Romania; dpantelica@yahoo.fr (D.P.); draceamariadiana@gmail.com (M.D.M.); 5 National Institute for Material Physics, 077125 Magurele, Romania; maraloiu@infim.ro (V.-A.M.); teoval@infim.ro (V.S.T.); 6 Academy of Romanian Scientists, 050094 Bucharest, Romania; 7 Research Center for Catalysts and Catalytic Processes, Faculty of Chemistry, University of Bucharest, Blv. Regina Elisabeta, No. 4-12, 030018 Bucharest, Romania

**Keywords:** lanthanum ferrite, pulsed laser deposition, water splitting

## Abstract

Tuning the intrinsic structural and stoichiometric properties by different means is used for increasing the green energy production efficiency of complex oxide materials. Here, we report on the formation of self-assembled nanodomains and their effects on the photoelectrochemical (PEC) properties of LaFeO_3_ (LFO) epitaxial thin films as a function of layer’s thickness. The variation with the film’s thickness of the structural parameters such as in-plane and out-of-plane crystalline coherence length and the coexistence of different epitaxial orientation—<100>SrTiO_3_//<001> LFO, <100>SrTiO_3_//<110> LFO and [110] LFO//[10] STO, as well as the appearance of self-assembled nanodomains for film’s thicknesses higher than 14 nm, is presented. LFO thin films exhibit different epitaxial orientations depending on their thickness, and the appearance of self-assembled nanopyramids-like domains after a thickness threshold value has proven to have a detrimental effect on the PEC functional properties. Using Nb:SrTiO_3_ as conductive substrate and 0.5 M NaOH aqueous solution for PEC measurements, the dependence of the photocurrent density and the onset potential vs. RHE on the structural and stoichiometric features exhibited by the LFO photoelectrodes are unveiled by the X-ray diffraction, high-resolution transmission electron microscopy, ellipsometry, and Rutherford backscattering spectroscopy results. The potentiodynamic PEC analysis has revealed the highest photocurrent density J_photocurrent_ values (up to 1.2 mA/cm^2^) with excellent stability over time, for the thinnest LFO/Nb:SrTiO_3_ sample, both cathodic and anodic behavior being noticed. Noticeably, the LFO thin film shows unbiased hydrogen evolution from water, as determined by gas chromatography in aqueous 0.5 M NaOH solution under constant illumination.

## 1. Introduction

The research activities focused on the reduction in the environmental pollutant energy sources and high-efficiency green energy production processes are at their highest level nowadays, having the main objective to fulfill the zero-emission international policies. The use of solar energy through photovoltaics and direct water photoelectrolysis is, in particular, of paramount importance for heavy energy consumption applications. Regarding the solar-driven photoelectrochemical water splitting for the hydrogen production process, there are still many difficulties to surpass in the development of high efficiency and stability photoelectrodes for achieving the required industrial application grade [1]. The production of hydrogen and oxygen via photoelectrochemical (PEC) water splitting is one of the most suitable ways for the conversion and storage of solar energy [2,3]. Mainly, in a PEC water splitting reaction, two electrochemical redox processes are involved: the hydrogen evolution reaction (HER) and the oxygen evolution reaction (OER). The HER is a classical two-electron transfer redox reaction (2H_2_O + 2e^−^ ⇄ H_2_ + 2HO^–^) occurring via the Volmer–Heyrowsky or Volmer–Tafel mechanism [4]. For OER, the mechanism is a four-electron transfer process (2H_2_O ⇄ O_2_ + 4H^+^ + 4e^−^), which leads to a more complicated half-reaction [5,6]. Moreover, depending on the nature of photoelectrodes, the reverse redox reactions, more specifically, the hydrogen oxidation reaction (HOR) and the oxygen reduction reaction (ORR), may become important competitors leading to a lowering of the overall water splitting efficiency. The HOR implies the oxidation of the molecular hydrogen H_2_ at cathode, using the same two-electron mechanism as HER. On the other hand, the ORR can take place at anode either by a direct or indirect four-electron transfer mechanism. The direct one can be a dissociative or associative process with the water formation. The indirect process involves first the generation of hydrogen peroxide followed by further reduction to water [7,8].

The inorganic perovskite materials have attracted a lot of attention lately for electronic and energy production-related applications. Till now, the perovskite-type materials have been extremely popular in photovoltaic applications, especially if one considers the reported results on the hybrid organic–inorganic compounds. The use of perovskite materials in solar energy harvesting by water splitting has a swift evolution lately, mainly due to the fact that the limitations of binary oxide semiconductors (TiO_2_, Fe_2_O_3_ or WO_3_), such as reduced band gap values or poor electrical charge transport properties seem to be unsolvable [9,10,11]. The possibility of tuning their intrinsic functional aspects such as dielectric, ferroelectric, or optical properties by employing strategies, such as epitaxial strain engineering or chemical doping, is the most interesting attribute of perovskite materials [12,13,14,15,16,17].

Good photocatalytic activities are reported for NaTaO_3_, CaTiO_3_, SrTiO_3_, and LaNiO_3_ perovskites under ultra-violet radiation, but the use of visible light is restricted by the wide band gap values [18,19,20,21,22]. Therefore, perovskites with smaller band gaps are of interest in photocatalysis for high water splitting efficiencies. BiFeO_3_ is one of the most studied multiferroics as it displays a band gap value of ~2.69 eV and increased photocatalytic activity [23]. Moreover, thin films of BiFeO_3_ have been reported to exhibit unassisted water splitting activity [12,24]. As mentioned previously, different strategies have been employed to enhance the functional properties of perovskite materials, especially associated to thin films [12,16].

In this context, lanthanum ferrite oxide LaFeO_3_ is a perovskite material having an even smaller band gap value than BiFeO_3_, more specifically ~2.07 eV, offering the possibility for better absorption in the visible range of the solar spectrum [25,26]. The photoelectrochemical properties of LFO photocathode prepared by screen-printed technique were studied by Celorrio et al., who have reported both cathodic and anodic photocurrent values [27]. In addition, the simultaneous production of both O_2_ and H_2_ using LaFeO_3_ powders was reported in other study [28]. The maximum of the obtained photocurrent value for LFO photoanode under simulated solar light is of ca. 0.8 mA/cm^2^ at higher potentials than 1 V vs. Ag/AgCl as Sora et al. have reported [29]. The unbiased spontaneous hydrogen generation by p-type LFO photoelectrode made by spray pyrolysis method has been reported by Pawar and Tahir [30]. The coral-like LFO nanostructure had yield up to 0.16 mA/cm^2^ at 0.26 V vs. RHE, with around 3.5% absorbed photon to current (APCE) efficiency.

Regarding the epitaxial thin film of perovskite materials, for increasing or tailoring a specific functional property, different strategies can be used. Thus, self-assembled nanostructured heteroepitaxial thin films of perovskite materials, but not limited to, have been reported to emerge due to imposed structural constrains coming from the lattice mismatch between the substrate and the film (epitaxial strain) or due to chemical effects such as doping [31,32,33]. The occurrence of self-assembled periodic structures or nanodomains, zones with different crystalline coherence lengths within the heteroepitaxial film, can lead to different changes in the intrinsic properties of the native materials, and controlling the intrinsic aspects of the formation and growth of such nanostructuring process will give the possibility to enhance certain functional properties [14].

Using PLD technique, Bi et al. have demonstrated the occurrence of self-assembled nanopyramid-like structures in the LFO epitaxial film deposited on SrTiO_3_ (001) substrates [34]. Adjusting specific experimental parameters such as substrate temperature and oxygen partial pressure, epitaxial films have been reported exhibiting a single orientation α-LFO(110)//STO(100) or a double orientation consisting of self-assembled nanopyramids β-LFO(001)//STO(100) embedded into a α-LFO(110)//STO(100) matrix. Within this paper, the authors have reported only on 300 nm thick LFO samples, the thickness effect over the nanopyramid self-assembling dynamics being overlooked. On the other hand, a detailed study concerning the effect of thickness on the photoelectrochemical properties of ultrathin films of LaFeO_3_/Nb:SrTiO_3_ was reported by May et al. [35]. Only samples of 10–25 nm thickness show cathodic photocurrents. The anodic photocurrent is observed for both thinner and thicker than 10 nm LFO films [35]. Noticeably, the nanostructures self-assembling effect was not mentioned in this paper. Moreover, to our knowledge, there are no reports on the photoelectrochemical properties of LFO thin films fabricated by PLD exhibiting such a self-assembled nanopyramid structures.

In this work, we present the structural dynamics of the self-assembled nanopyramid-like structures within the epitaxial LFO thin films deposited by PLD. The occurrence of the nanopyramid-like structures and the effects over the photoelectrochemical properties of LFO/Nb:SrTiO_3_ thin films as a function of layer’s thickness are presented. The variation with the film’s thickness of the structural parameters such as in-plane/out-of-plane crystalline coherence lengths and the coexistence of different epitaxial orientations within the films in conjunction with the PEC efficiency are discussed.

## 2. Materials and Methods

### 2.1. Thin Films Deposition

The LFO thin films were deposited by pulsed laser deposition (PLD) starting from a ceramic target of LaFeO_3_ on pristine Nb:SrTiO_3_ (STON) substrates. The doping of SrTiO_3_ with Nb was performed for electrical purposes, because a conductive substrate is required for photoelectrochemical (PEC) measurements. An ArF excimer laser (193 nm) with 5 Hz repetition rate was used for fabrication of samples. The substrates were heated at 50 °C/min up to 750 °C, and the deposition process was performed in oxygen gas (0.6 mbar partial pressure). All samples were manufactured using the same laser fluence (2.1 J/cm^2^). The films thickness was varied in 14–200 nm range.

### 2.2. Characterization and Photoelectrochemical Measurements

The investigation of the sample optical properties was performed using reflection type spectroscopic ellipsometry measurements, on a Woollam Variable Angle Spectroscopic Ellipsometer (VASE) system equipped with a high-pressure Xe discharge lamp, in the spectral range of 1–5 eV from the near IR to the UV. The change of the polarization state of linearly polarized light due to reflection at the surface was measured. In order to obtain refractive index and extinction coefficients for the thin films, WVASE32 software (VASE, J.A. Woollam Co., Inc., Lincoln, NE, USA) was used for fitting and extracting the useful data from complex multilayer response. Standard ellipsometry measurements have been carried out at a fixed angle of incidence in the spectral range of 300–1200 nm.

High-resolution X-ray diffraction conducted on a PANalytical X’Pert MRD system (Almelo, The Netherlands) equipped with a fixed Cu anode and a four-bonce double crystal Ge (220) placed in the incident beam path to generate a monochromatic X-ray beam (λ = 1.54056 Å) was used for the investigation of the thin films structure. Additional structural data were acquired from high-resolution transmission electron microscopy (HR-TEM) analysis performed on a Jeol ARM 200F microscope (Joel, Tokyo, Japan). The high- and low-magnification transmission electron microscopy (TEM, HR-TEM) analyses in cross-section (XTEM) have been performed using a Jeol ARM 200F electron microscope. The cross-section specimens were prepared using the classical tripod mechanical thinning and polishing method and finally by ion thinning in a Gatan device (Gatan Inc., Pleasanton, CA, USA).

The elemental composition of the LFO samples was determined using Rutherford backscattering spectrometry (RBS) (High Voltage Engineering Europa B.V. —HVE, Amersfoort, Netherlands) analysis with a collimated 3.043 MeV He^2+^ beam, delivered by the 3 MV Tandetron Cockcroft–Walton accelerator, at the IBA beam-line. The sample was tilted at 70 relative to the beam direction. The alpha particles were detected with a passivated, ion implanted silicon detector, placed at 165° with respect to the incident beam direction. The diameter of the detector was 8 mm, and the distance between the sample and the detector was 175 mm, resulting in a solid angle of 1.641 msr. The energy resolution of the detector was around 17 keV.

The photoelectrochemical measurements were performed using a three-electrode system coupled with a quartz cell. The counter electrode was a helicoidal Pt wire, the reference electrode was Ag/AgCl (3.5 M KCl), and the working electrode was composed of the LFO/STON thin film itself. The electrical connection was obtained on the back of the STON substrates using conductive wire and silver paste. The back and sides of the samples were encapsulated into an insulator non-corrosive epoxy resin, in order to avoid any electrical short circuit of the substrate in contact to electrolyte solutions. The measurements were performed in 0.5 M NaOH solution (pH = 13.7). The laser diode used for irradiation emits at 405 nm (5 mW output power). To avoid the effect of concentration polarization, a small scanning rate (5 mV/s) was used for linear sweep voltammetry (LSV) measurements. The unbiased hydrogen evolution from water was determined by gas chromatography in aqueous 0.5 M NaOH solution under a constant illumination. The generated hydrogen gas was quantitatively analyzed using a Shimadzu Nexis GC-2030 gas chromatograph (GC) (Shimadzu, Kyoto, Japan) equipped with a ShinCarbon ST micro-packed column (Restek Corporation, Bellefonte, PA, USA) and a barrier ionization discharge (BID) detector; for supplementary details, see Supplementary Materials file.

## 3. Results

As mentioned, LFO thin films with thickness values between 14 and 200 nm have been obtained, the thickness values being extracted from the spectrometric ellipsometry measurements and transmission electron microscopy in cross-section. The photoelectrochemical properties of LFO/STON thin films are presented in correlation with the fine structural changes and the appearance of self-assembled nanopyramid nanostructures as a function of the film’s thickness value. The structural features of the samples were revealed by both high-resolution XRD and XTEM, different thickness value LFO/STON thin films being investigated.

### 3.1. XRD Measurements

**Figure 1:**
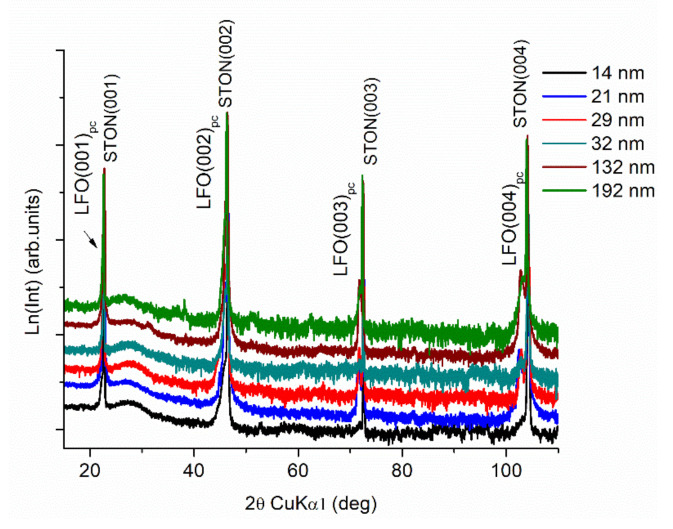
The XRD patterns of LFO films with different thicknesses deposited on STON.

High-resolution XRD measurements of the LFO films reveal fully epitaxial grown films, all of them exhibiting only (00*l*) reflections (Figure 1). LFO belonging to the family of orthoferrites, is a distorted perovskite structure at room temperature. It crystallizes in an orthorhombic symmetry (a = 5.557 Å, b = 5.5652 Å, and c = 7.8542 Å), space group Pbmn [34,36]. When grown on cubic (001) oriented substrate, such as STO(N)(001), two film orientations, LFO(110)||STON(100) (the α domain) and LFO(001)||STON(100) (the β domain) show similar lattice mismatch. Consequently, the LFO diffraction peaks originating from the (*l*, *l*, 0) and (0, 0, 2*l*) planes of the α and β domains, respectively, merged in singular peaks [34,37]. For convenience, we used a pseudo-cubic notation as reported in [37,38]. The position of XRD (00*l*)_pc_ reflections give the out-of-plane lattice parameter. The perpendicular coherence length (L_┴_), which is to be connected to the nominal film thickness; the heterogeneous strain perpendicular to the substrate (ε_┴_); the lateral coherence length (L_||_); and the mean mosaic tilt angle (α_tilt_) were calculated using Williamson–Hall type approaches, which were comprehensively described in our previous articles [14,16]. The Williamson–Hall method aimed to separate the contributions to the peak broadening of the finite size of a crystalline domain in the sample and the heterogeneous strain owing to lattice defects by comparing the peak widths of reflections of successive orders [39]. It had been proposed initially for polycrystalline materials [39]. Extended to films, the approach implies a “mosaic” model of the films, by assuming that epitaxial films grown on lattice-mismatched substrates consist of oriented mosaic blocks that coherently scatters X-rays. The dimension of the mosaic blocks in the growth direction is the perpendicular coherence length, L_┴_, and in the growth plane is the lateral coherence length, L_||_. The mosaic blocks can also be slightly miss-oriented out of the sample plane, i.e., tilt angle, α_tilt_, or within the sample plane, i.e., twist angle [40,41]. It was mainly utilized for epitaxial III-nitrides films [42,43] or columnar ZnO films [44]. Analyzing of rocking curve (ω-scans) broadening as a function of diffraction vector allows independent determination of mosaic tilt angle and lateral coherence length, while the same type of analysis applied to the broadening along the ω-2θ direction enables independent deduction of heterogeneous strain and the perpendicular coherence length [40,41]. For three films, the in-plane lattice parameters were calculated from off-axis diffraction scans at different tilt ψ angles for the four azimuthal angles. The structural data derived from the XRD measurements along with the film thickness, evaluated through spectrometric ellipsometry, and validated by HR-TEM are gathered in Table 1.

**Figure 2:**
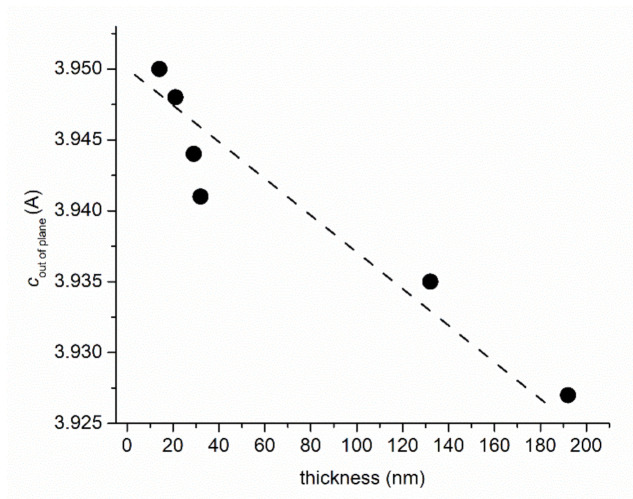
Variation of the thickness of the LFO films (determined via spectroscopic ellipsometry) with the out-of-plane lattice parameter (determined by XRD).

A decrease in the out-of-plane lattice constants with increasing thickness is observed (Figure 2). This corresponds to a decrease in the lattice expansion compared to bulk (pseudo)cubic LFO (standard LaFeO_3_, a = 3.926 Å, ICDD card no. 01-075-0541) from 6.1% to almost 0% for the thickest film. This stress relaxation is associated by some authors to the deposition conditions, increasing oxygen pressure causing a decrease in oxygen vacancies [34,45] or the same effect of decrease in oxygen vacancies by thermal annealing in oxygen [46]. We observed the same trend in our work devoted to LFO film deposited under different oxygen partial pressure [47]. Since the films are deposited under the same thoroughly controlled partial oxygen pressure (0.6 mbar), a different explanation proposed by Scafetta and May in a more recent study is more plausible [38]. They show that Fe deficiency, hence Fe vacancies, induced an increase in out-of-plane lattice parameter of LFO films deposited on STO. The same effect of expansion of the out-of-plane lattice parameter due to the presence of lattice-distorting defects, such as vacancies is reported for rare-earth nickelates [48].

**Figure 3:**
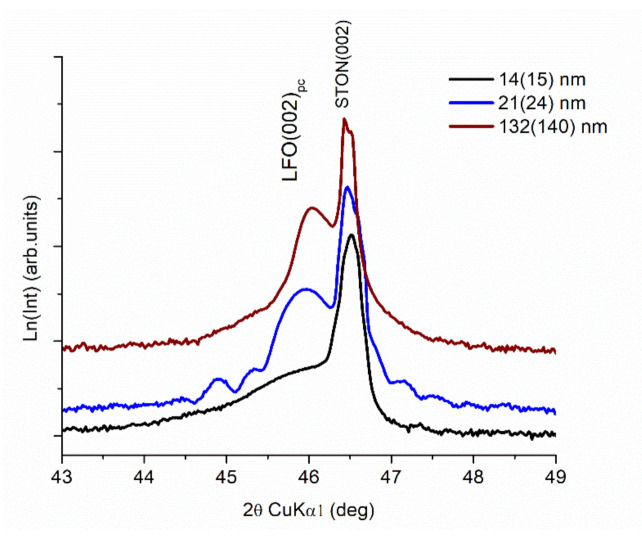
The θ–2θ patterns for thin films around the (002)_pc_ reflection for LFO/STON thin films with different thicknesses.

In spite of the stress induced by the expansion of the lattice, the quality of films is quite good with sharp diffraction peaks and discernible Laue oscillation, in particular, for the 14–21 nm LFO film (Figure 3), in line with previously reported results [47]. This is relevant for an excellent interface quality of the film [49]. The thicker film exhibits no Laue fringes. The good interface quality is also sustained by the in-plane lattice parameters, which are almost equal to that of the STON substrate (a = 3.905 Å) for the three films with different thicknesses for which they were determined. The coherence perpendicular length that should be proportional to the films’ thickness is significantly smaller for the thicker films, indicative of a more defective structure, in spite of the relaxation occurring along the growth axis. The microstrain ε _┴_, estimating defects along the growth direction, is roughly proportional to the thickness of the films: smaller for the thinner ones and larger for the thicker ones. However, there is not a perfect linearity between thickness and microstrain due to the limitation of the “mosaic block” model and the fact that the microstrain, ε _┴_, includes only the out-of-plane strain and not the other two in-plane strain components. The mosaicity of the LFO expecting the thickest LFO/STON film (≈193 nm) is relatively good as mean mosaic tilt angle (α_tilt_) and lateral coherence length (L_||_) values reveal. In particular, for the 14 and 21 nm LFO films, the L_||_ and the α_tilt_ values are indicative of an excellent ordering along the growth direction. However, the measured rocking curves are not a typical Gaussian peak commonly observed. We already reported on this aspect regarding the LFO films deposited at different oxygen pressures [47].

**Figure 4:**
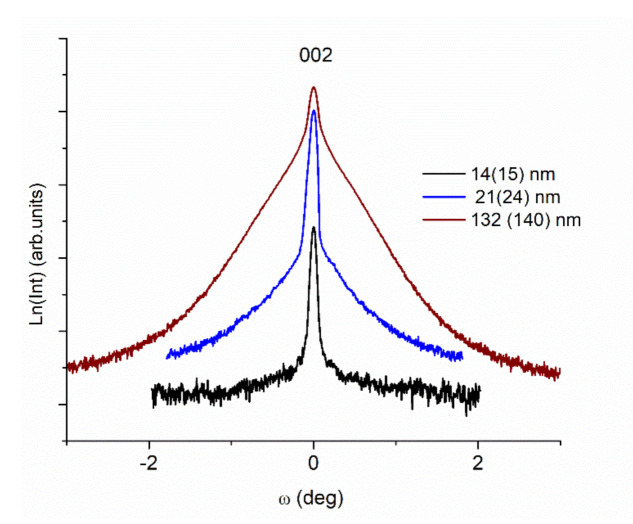
The X-ray rocking curves ω-scan measurements for LFO thin films with different thickness values.

Figure 4 presents the rocking curves of three films around the (002)_pc_ reflection with different thickness values. The intensity of rocking curve is plotted in logarithmic scale highlighting better the two-contributing curves feature occurring for thicker samples. This effect is to be explained by the presence of two regions having different degrees of orientation, a highly oriented one and more distorted one [50,51,52]. The effect is noticeable for the thicker films, e.g., a 132 nm LFO film.

### 3.2. Photoelectrochemical (PEC) Measurements

**Figure 5:**
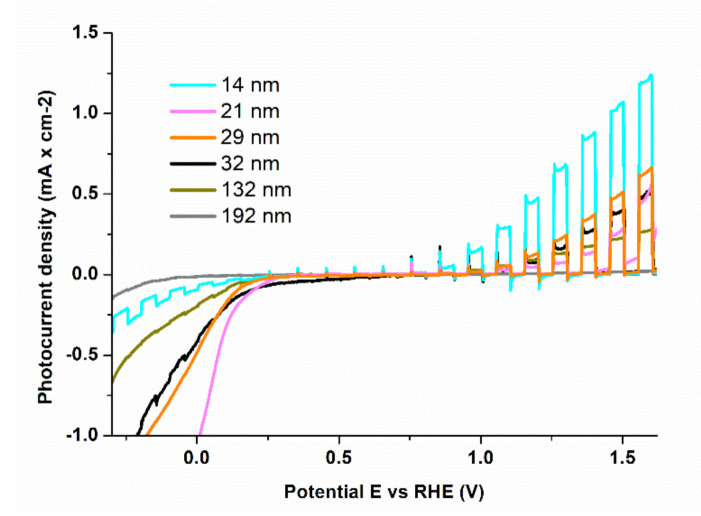
Potentiodynamic measurements under chopped irradiation on LFO/STON thin films with different thickness values in alkaline 0.5 M NaOH electrolyte.

The photoelectrochemical water splitting performance of the LFO/STON thin films as a function of film’s thickness has been determined by chopped potentiodynamic and potentiostatic measurements using 405 nm laser light (5 mW output power), in the −0.5–1.6 V vs. RHE potential range. The potentiodynamic measurements, presented in Figure 5 for LFO/STON thin films with thicknesses from 14 to 192 nm, clearly show the higher values of photocurrent density J_ph_ for the thinnest LFO/STON samples, up to J_ph_ = 1.23 mA/cm^2^ at 1.6 V applied potential vs. RHE being recorded for the 14 nm sample. For LFO/STON thin films with thicknesses above 32 nm, the anodic photocurrent has higher values toward the positive potential, while for the thinnest LFO/STON samples, both photocathodic and photoanodic photocurrents have been measured. The cathodic photocurrent is observed below 0.35 V vs. RHE applied potential; above this potential value the photocurrent switches to anodic with clear n-type characteristics for higher RHE values. Indeed, there are several reports on the fact that LFO nanostructures exhibit both anodic and cathodic behavior as a function of the used experimental methods [27,35,53].

For LFO photoanodes, Peng et al. reported the presence of cathodic photocurrent with p-type characteristics under chopped visible light illumination above 420 nm and in the negative potential less than −0.33 V vs. Ag/AgCl [54]. For ultrathin LFO/STO obtained by PLD, May et al. have reported a strong dependency of the cathodic photocurrent on the film thickness, which was observed only for samples of 10–25 nm thickness, while the anodic photocurrent was observed for both thinner and thicker than 10 nm LFO/STON films [35].

**Figure 6:**
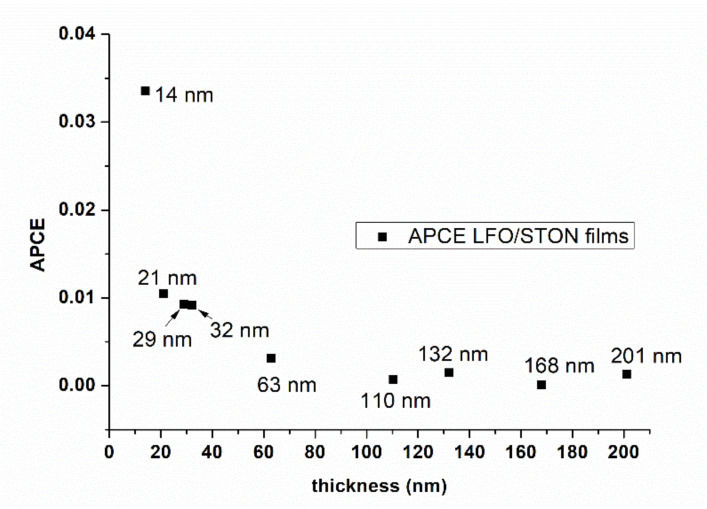
APCE efficiency as a function of films thickness.

Most importantly, for a direct evaluation of the unassisted LFO thin films water splitting efficiency, unbiased hydrogen production performance measurements have been performed on the thinnest LFO/STON samples. The measurements have been done by gas chromatography technique in aqueous 0.5 M NaOH solution under a constant illumination, without any applied external bias—see Figure S1, Supplementary Materials file. The water splitting test has been performed for more than 4 h, up to 6.5 µmoles/cm^2^ of hydrogen being produced. Related data and the interconnection with the PEC efficiencies are still under way. The absorbed photon to current efficiencies dependence of the LFO/STON thin films thickness value at 1.6 V vs. RHE, presented in Figure 6, were calculated using the following formula:

$$\mathrm{APCE}=\frac{J(\mathrm{mA}\cdot {\mathrm{cm}}^{-2})\times 1239.8\;\left(V\cdot \mathrm{nm}\right)}{P_{\lambda }(\mathrm{mW}\cdot {\mathrm{cm}}^{-2})\times \lambda \left(\mathrm{nm}\right)\times A}$$

where J is the photocurrent density, P_λ_ is the incident power density, λ is the wavelength of the irradiation source, and A is the absorptance calculated as shown below from the extinction coefficients (k) measured by spectroscopic ellipsometry and the film thickness (t) calculated by XTEM:

$$A=1-e^{-\frac{4\pi k\cdot t}{\lambda }}$$

The incident power at the films’ surface was 883 mW/cm^2^, the irradiated spot size being 0.5 mm^2^.

To further understand the potentiodynamic PEC results, the fine structural, optical, and stoichiometric changes induced into the films during the PLD growth have been investigated, high-resolution transmission electron microscopy in cross-section (XTEM), spectrometric ellipsometry (SE), and Rutherford backscattering techniques being employed. 

### 3.3. High-Resolution Electron Microscopy in Cross-Section (XTEM)

For the XTEM analysis, three different thickness values of LFO/STON films have been chosen: 14, 21, and 132 nm. In these three cases, different morphologies were observed, the occurrence of the nanopyramid-like structures being revealed.

**Figure 7:**
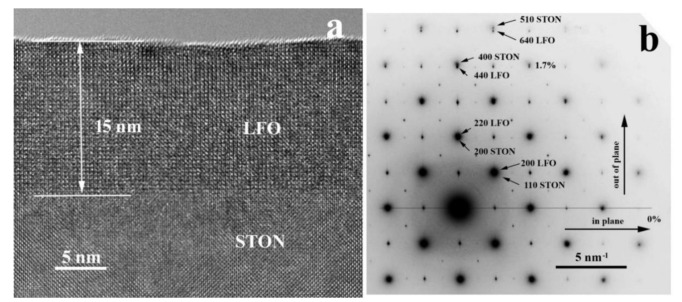
HRTEM images of LFO/STON sample with 15 nm thickness (**a**), corresponding to SAED pattern of the LFO film and the STON substrate in [100] orientation (**b**).

Figure 7 shows the LFO/STON epitaxial film with a thickness of about 15 nm in the [100] crystallographic orientation. The corresponding SAED pattern show the perfect alignment of the two lattices in the interface plane and a mismatch of about 1.7% out of plane. This means that the LFO is compressed in the interface plane and expanded out of plane, as the lattice constant of the cubic LFO is 0.3926 nm (ICDD card no. 82-1958) and is larger with only about 0.6% compared with the 0.3903 nm STON lattice constant.

**Figure 8:**
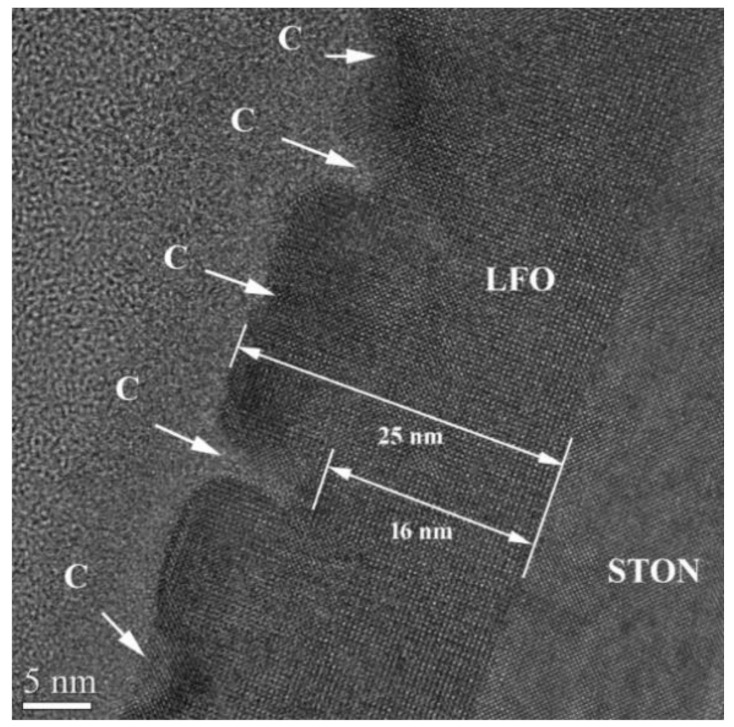
High-resolution XTEM image of the LFO/STON film with the thickness of about 25 nm in the [100] crystallographic orientation.

Figure 8 shows a HRTEM image of the LFO/STON film with 25 nm thickness. Several crevasse-like defects (denoted C) are observed on the film surface. These defects penetrate about 8–9 nm into the film. The LFO/STON near interface region of the film shows no defects. This morphology indicates that the film thickness limit without relaxation is about 17–20 nm. From this level, some defects appear, which in this case, look like crevasses. These crevasses are several nanometers thick and look to be filled with an amorphous content.

**Figure 9:**
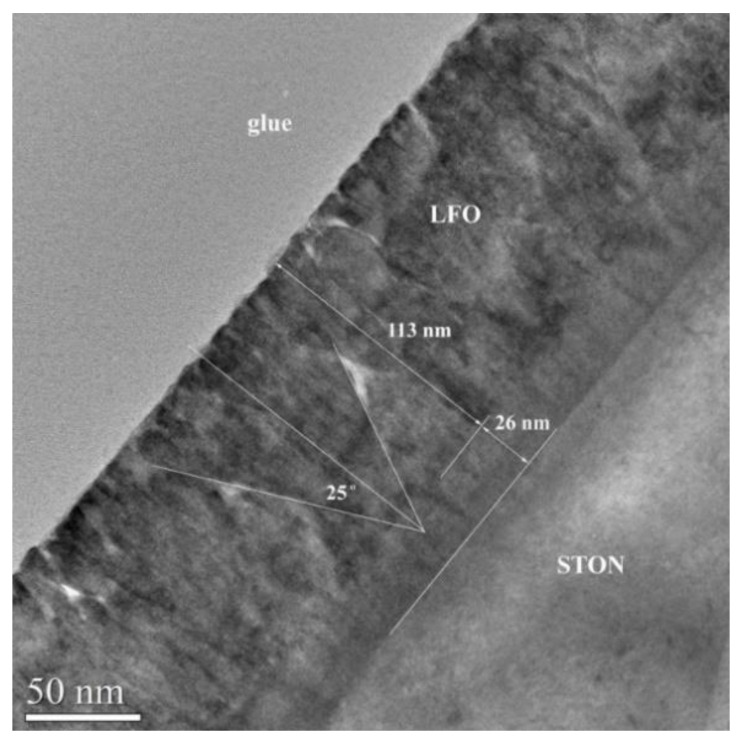
Low-magnification XTEM image of the LFO/STON film with the thickness of about 132 nm.

It is worth to mention that the occurrence of the nanopyramid-like structures is clearly visible (see Figure 9) for the thickest LFO/STON sample of 140 nm. At a closer look, two distinctive regions are present here: a less-defective 20–25 nm region close to the LFO/STON interface and the region of the rest of the film toward the surface exhibiting a succession of 50 nm at the baseline nanopyramid-like structure developed between the top of the near-interface region.

**Figure 10:**
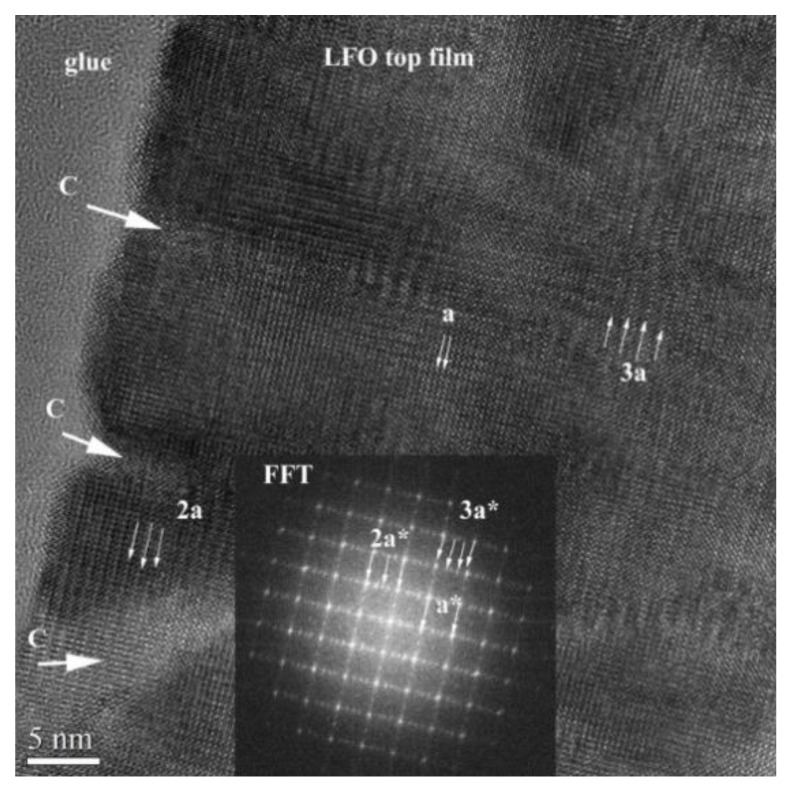
High-resolution XTEM image of the top of the LFO film. Inset is the FFT pattern performed on the image area. (a* is denoted as the presence of the superstructure in FFT pattern analyses of the zone defined by 2a and 3a).

Figure 10 shows the disordered lattice of the nanopyramid-like structure near the film surface. The top crevasses (designated with C) are continued into the film volume and form the limits of the nanopyramid structure. These limits are several nanometers in thickness and look to be amorphous. The FFT (fast Fourier transform) pattern shows the appearance of multiple nanodomains with superstructures exhibiting a doubled or tripled lattice parameter value. Here, the *a-*constant (0.3926 nm) is the quasi-cubic LFO parameter. The *c* parameter of the orthorhombic LFO is *c = 2a,* but the 3*a* interval represents the presence of a superstructure.

**Figure 11:**
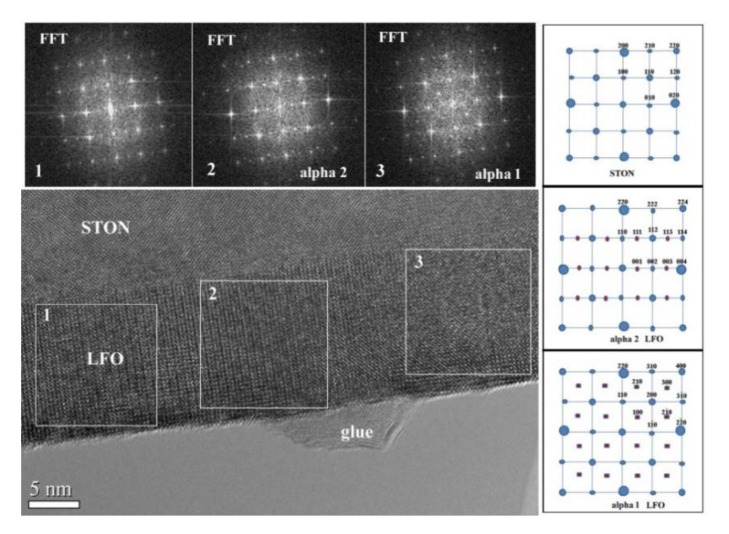
HRTEM of 15 nm LFO/STON film showing the two different orientations of the [001] LFO direction respective to the (100) STON substrate plane: alpha 1, the [001] LFO is parallel to the image normal (microscope axis), and alpha 2, the [001] LFO is in the image plane.

For the thinnest 14 nm LFO/STON sample, there are no signs of formation of any incipient crevasse at the surface of the film. The FFT pattern analyses reveal the presence of the alpha 1 and alpha 2 epitaxial orientations for the LFO/STON film (see Figure 11). The size of these regions is of the order of 20–30 nm in the film plane, and probably is responsible for some relaxation, without destabilizing the epitaxial ordering.

The θ–2θ and ω-scan X-ray rocking curves shape as well as the mean mosaic tilt angle (α_tilt_) and lateral coherence length (L_||_) values are validated by the HRTEM analysis, which reveals the formation of disoriented domains with cracks/crevasses and defects at the surface and in volume of the films with the nanopyramid-like appearance clearly visible for thick samples.

### 3.4. Optical Properties: Spectrometric Ellipsometry (SE)

The optical properties of LFO/STON thin films with different thicknesses, in terms of refractive index (n), extinction coefficients (k), and the corresponding dielectric function (ε_1_ and ε_2_) thickness dependence, were determined using a modified Levenberg–Marquardt algorithm to fit the ellipsometry spectra. The three-medium structural model used in our calculation was composed of a semi-infinite substrate, LaFeO_3_ thin film, the rough layer, and air ambient. Before the PLD deposition, the single-crystals of niobium-doped SrTiO_3_ used as substrate were measured in the same range of wavelength, and the dielectric function of semi-infinite substrates were extracted by fitting the experimental data point-by-point.

The fitting procedure was done in three steps. First, the film thickness and surface roughness were determined by fitting the experimental data of Ψ and Δ using the Cauchy model in the 700–1700 nm spectral range where the thin film was supposed to be optical transparent. The surface roughness layer was consisted to be a mixture of the film and voids in proportion of 50%:50%. The dielectric function of rough top layer was calculated by the Bruggeman effective medium approximation (EMA) [55]. In the second step, the dielectric function (ε_1_, ε_2_) in the optical range of frequency of LaFeO_3_ thin films were modelled by a sum of four Gaussian oscillators fitting the experimental data on the entire measured spectra (1–5 eV) [56]. After the dielectric function of LaFeO_3_ thin film being obtained, the final adjustment of thickness and roughness was done by fitting all “parameters” used in our optical model, obtaining in this way, the lower value of MSE. The fitting parameters used to obtain the dielectric function spectra for all analyzed samples are listed in Table S1 in the Supplementary Materials.

**Figure 12:**
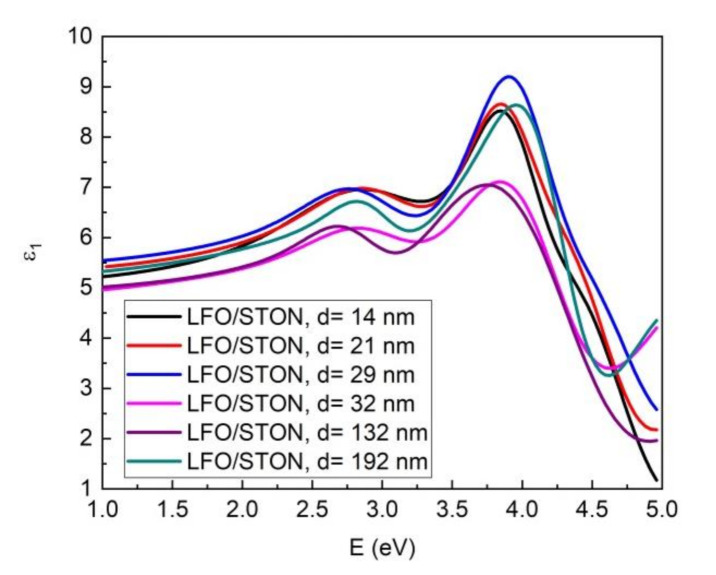
The dispersion of the real part of dielectric function ε_1_ for LFO/STON thin films.

**Figure 13:**
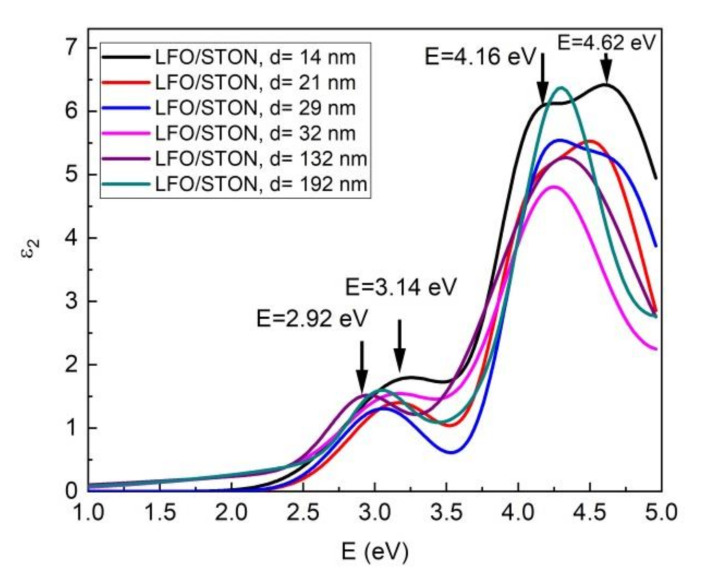
The dispersion of the imaginary part of dielectric function ε_2_ for LFO/STON thin films.

The above proposed optical model was used only for samples of LFO with the lower thickness, and for thicker samples, the number of Gauss oscillators was changed. In this case, the best fit was obtained with three oscillators. The dispersion of the real and imaginary part of dielectric function, in the optical frequency regime, for LFO thin films are presented in Figure 12 and Figure 13.

For the imaginary part ε_2_ of dielectric constants, which is directly related with optical absorption, the dispersion curve shows a few peaks at higher energy. In the 1–2 eV spectra (620–1240 nm), most of LFO samples are optically transparent. For the thinnest sample (14 nm), the first peak is located at energy of 3.14 eV and is followed by two more peaks at 4.16 and 4.62 eV. This optical behavior of LFO thin films as a function of cation stoichiometry was well described by Scafetta et al. According to their findings, from the spectral shape of ε_2_ and peaks position of our films, it is clear that the 14 nm LFO/STON sample has a La deficiency [38]. As the thickness of the samples increases, the spectral shape is changed. The first peak is shifted to lower energy from 3.14 to 2.92 eV for thicker sample. The second and third peaks of ε_2_ are also shifted and the spectral shape is changed, indicating a different cations ratio for the samples with the thickness higher than 14 nm. For the 21 and 29 nm LFO/STON samples, the changed position and shape of the second and third ε_2_ peaks are obvious, therefore implying a different Fe/La cation ratio into the films. The cation ratio modification is continuing for thicker samples. Thus, for the samples with thickness higher than 30 nm, the ε_2_ spectra shape is dramatically changed in the 3.5–5 eV energy range, and this behavior is directly related to crystalline state, the LFO films with high thickness have a higher disorder in crystalline structure, as the XTEM and XRD analysis revealed.

### 3.5. Stoichiometric Investigations: Rutherford Backscattering

Rutherford backscattering spectroscopy (RBS) using 2 MeV α particles is a standard technique for the quantitative and nondestructive analysis of thin films, usually used for depth profiling of heavy elements in a matrix containing lighter elements.

**Figure 14:**
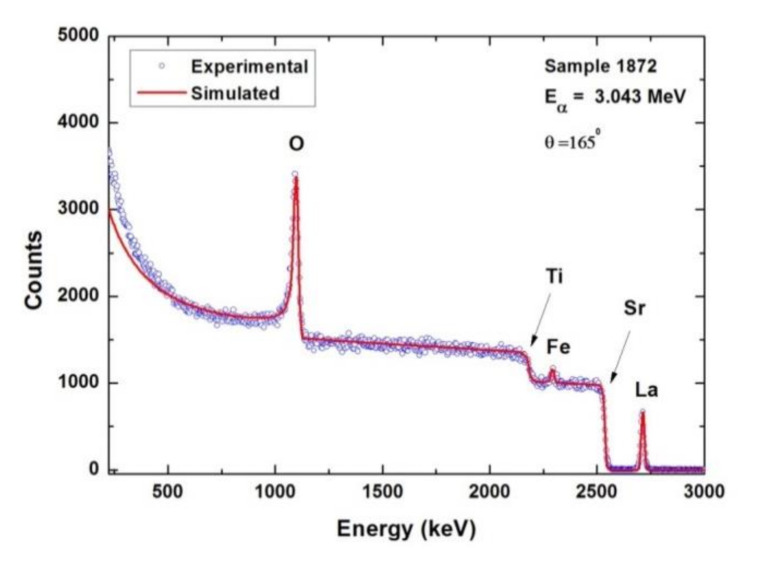
Representative RBS spectrum and simulation result recorded on 14 nm LFO/STON sample.

To confirm the optical data, compositional investigations have been performed, namely, Rutherford backscattering. The elemental composition of the LFO/STON samples was determined using Rutherford backscattering spectrometry (RBS) analysis in high vacuum (10–6 mbar). Counting rates were always kept small enough in order to have a negligible dead time during the measurements. The elemental concentrations and thicknesses were inferred using the simulation code SIMNRA by fitting the RBS experimental spectrum [57]. The result of the simulation is presented with a continuous line (Figure 14). The RBS analysis is summarized in Table S2 in the Supplementary Materials. The error of RBS measurements is estimated between 1% and 2%.

The change in the cation ratio Fe/La as a function of LFO film’s thickness, observed from the optical data provided by the ellipsometry is confirmed by the RBS analysis, within the RBS technique limits. The La-deficient samples are the thinnest ones, while the Fe-deficiency appears for samples with high thickness. In accord with the optical data, the LFO/STON films with thickness between 14 and 32 nm are considered nominally stoichiometric within the RBS limits.

## 4. Conclusions

The PEC performance as a function of thickness of epitaxial LFO thin films deposited by PLD, in correlation with fine optical, structural, and stoichiometric features, has been presented. The appearance of self-assembled nanopyramid-like structures within the epitaxial LFO thin films, along with significant changes in cation Fe/La ratio and implicitly in the functional PEC properties have been determined. Different structural features have been noticed for LFO/STON thin films, from a single orientation α-LFO(110)//STO (100) to double orientation consisting of self-assembled nanopyramids β-LFO(001)//STO(100) embedded into a α-LFO(110)//STO(100) matrix. From the PEC measurements performed on LFO/STON with thicknesses above 32 nm, the anodic photocurrent has higher values toward the positive potential, while for the thinnest LFO/STON samples, both photocathodic and photoanodic photocurrents have been measured. Most importantly, for a direct evaluation of the unassisted LFO thin films water splitting efficiency, unbiased hydrogen production performance measurements have been performed on the thinnest LFO/STON samples. The water splitting tests have been performed for more than 4 h, up to 6.5 µmoles/cm^2^ of hydrogen being spontaneously produced, as determined by gas chromatography. The occurrence of the nanopyramid-like structures has been proven to have a detrimental effect over the photoelectrochemical properties of LFO/Nb:SrTiO_3_ thin films.

## Figures and Tables

**Table 1:** The structural data of the LFO films. The thickness is determined by spectrometric ellipsometry. For three films, the thickness values were measured through high-resolution electron microscopy in cross-section (XTEM); the values are added in brackets.

D (nm)	Structural Data					
	a _out-of-plane_ (nm)	a _in-plane_ (nm)	L_┴_ (nm)	ε_┴_ Microstrain (%)	α_tilt_ (°)	L_II_ (nm)
14(15)	3.950	3.899	13	0.01	0.095	512
21(24)	3.948	3.902	35	0.06	0.09	752
29	3.944	n.det	32	0.02	0.13	350
32	3.941	3.904	33	0.25	0.14	151
132(140)	3.935	n.det	71	0.14	0.18	195
192	3.927	n.det	127	0.40	1.22	38

n.det: not determined.

## Data Availability

Data is contained within the article or supplementary materials.

## Supplementary Materials

The following are available online at https://www.mdpi.com/article/10.3390/nano11061371/s1, Figure S1: The photocatalytic hydrogen generation in time for the LFO/STON sample having 32 nm thickness. Table S1: The fitting parameters used to obtain the dielectric function spectra for all analyzed samples, Table S2: The cation composition and ratio for three different thicknesses LFO/STON thin films.
