# Supplementary material for: Thickness-Dependent Photoelectrochemical Water Splitting Properties of Self-Assembled Nanostructured LaFeO3 Perovskite Thin Films
Source: Nanomaterials (Basel). 2021 May 21;11(6):1371. doi: 10.3390/nano11061371 (PMC8224280; doi:10.3390/nano11061371)
Supplement: Supplementary file 1 [file nanomaterials-11-01371-s001.zip › nanomaterials-1225349-supplementary.pdf]

Supplementary Materials

# Thickness-Dependent Photoelectrochemical Water Splitting Properties of Self-Assembled Nanostructured $LaFeO_3$ Perovskite Thin Films


**Florin Andrei [1,2], Valentin Ion [1], Ruxandra Bîrjega [1], Maria Dinescu [1], Nicoleta Enea [1,3], Dan Pantelica [4], Maria Diana Mihai [4], Valentin-Adrian Maraloiu [5], Valentin Serban Teodorescu [5,6], Ioan-Cezar Marcu [2,7,*] and Nicu Doinel Scarisoreanu [1,*]**

[1] National Institute for Laser, Plasma and Radiation Physics, 077125 Magurele, Romania; florin.andrei@inflpr.ro (F.A.); valentin.ion@inflpr.ro (V.I.); ruxandra.birjega@inflpr.ro (R.B.); maria.dinescu@inflpr.ro (M.D.); nicoleta.enea@inflpr.ro (N.E.)
[2] Laboratory of Chemical Technology & Catalysis, Department of Organic Chemistry, Biochemistry & Catalysis, Faculty of Chemistry, University of Bucharest, Blv. Regina Elisabeta, No. 4-12, 030018 Bucharest, Romania
[3] Faculty of Physics, University of Bucharest, 077125 Magurele, Romania
[4] Horia Hulubei National Institute for R&D in Physics and Nuclear Engineering, 077125 Magurele, Romania; dpantelica@yahoo.fr (D.P.); draceamariadiana@gmail.ro (M.D.M.)
[5] National Institute for Material Physics, 077125 Magurele, Romania; maraloiu@infim.ro (V.-A.M.); teoval@infim.ro (V.S.T.)
[6] Academy of Romanian Scientists, 050094 Bucharest, Romania
[7] Research Center for Catalysts and Catalytic Processes, Faculty of Chemistry, University of Bucharest, Blv. Regina Elisabeta, No. 4-12, 030018 Bucharest, Romania
* Correspondence: ioancezar.marcu@chimie.unibuc.ro (I.-C.M.); nicu.scarisoreanu@inflpr.ro (N.D.S.); Tel.: +40-21-3051464 (I.-C.M.); +40-74-3147427 (N.D.S.)


**Unbiased Hydrogen Production Measurements**

The photocatalytic hydrogen production measurements of LFO/STON thin films were performed in a glass cell (total volume of ca. 35 mL) with a quartz window for irradiation. The sample was immersed in 15 mL of NaOH aqueous solution under magnetic stirring. The irradiation of the sample was realized using a laser diode emitting at 405 nm. The generated hydrogen gas was quantitatively analyzed using a Shimadzu Nexis GC-2030 gas chromatograph (GC) equipped with a ShinCarbon ST micro-packed column and a barrier ionization discharge (BID) detector. High purity helium was used as carrier gas. The gas sample was injected into the GC using a HAMILTON syringe with a volume of 250 μL. The GC parameters used to record chromatograms were: the injector pressure, temperature and split ratio 250 kPa, 150 °C, and 1:5, respectively, the column temperature, 35 °C and the detector temperature and discharge gas flow, 280 °C and 100 mL/min, respectively.

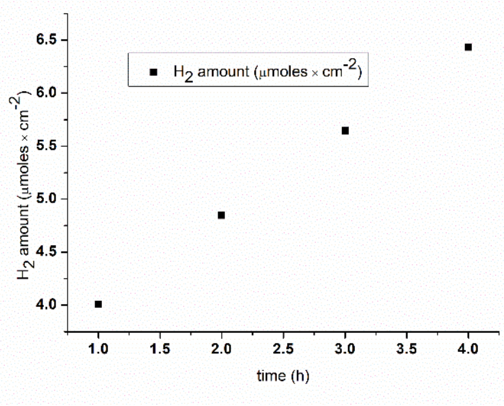


**Figure S1.** The photocatalytic hydrogen generation in time for the LFO/STON sample having 32 nm thickness.

## Optical Properties: Spectroellipsometry Data

**Table S1.** The fitting parameters used to obtain the dielectric function spectra for all analysed samples.

| Thickness (nm) | Roughness (nm) | An | En (eV) | Br (eV) | MSE |
|---|---|---|---|---|---|
| 14.1 | 0.3 | 1.7421 | 3.2072 | 0.9651 | 5.231 |
| | | 2.6678 | 4.0374 | 0.4826 | |
| | | 6.3246 | 4.6299 | 1.0706 | |
| | | 8.0825 | 8.6943 | 2.9356 | |
| 21.2 | 7.4 | 1.3958 | 3.1603 | 0.7928 | 1.933 |
| | | 2.5416 | 4.0138 | 0.4921 | |
| | | 5.3508 | 4.5435 | 0.8439 | |
| | | 11.757 | 9.1872 | 3.3178 | |
| 29.2 | 7.6 | 1.3066 | 3.0576 | 0.7310 | 1.561 |
| | | 3.289 | 4.1173 | 0.5497 | |
| | | 4.9815 | 4.6746 | 0.9063 | |
| | | 11.642 | 9.3884 | 3.327 | |
| 31.3 | 1.2 | 0.2882 | 2.6422 | 2.269 | 1.335 |
| | | 1.1577 | 3.1271 | 0.7818 | |
| | | 4.0399 | 4.2221 | 0.8237 | |
| | | 6.8136 | 7.2933 | 3.3636 | |
| 132.1 | 0.5 | 1.0662 | 2.9212 | 0.5608 | 2.044 |
| | | 4.4495 | 4.3144 | 1.09 | |
| | | 4.546 | 11.99 | 9.7818 | |
| 192.8 | 9.1 | 0.9059 | 3.0226 | 0.4734 | 2.183 |
| | | 4.6067 | 4.2825 | 0.6108 | |
| | | 6.3301 | 8.2568 | 5.8434 | |

## Compositional Analysis: Rutherford Backscattering Spectrometry (RBS) Analysis

**Table S2.** The cation composition and ratio for three different thicknesses LFO/STON thin films.

| Sample | Composition | | Fe/La |
|---|---|---|---|
| 14 nm LFO/STON | Fe<br>0.28 (±3.5%) | La<br>0.23 (±1%) | 1.217 |
| 29 nm LFO/STON | Fe<br>0.206 (±3%) | La<br>0.196 (±0.7%) | 1.051 |
| 32 nm LFO/STON | Fe<br>0.163 (±3%) | La<br>0.224 (±1%) | 0.727 |
